# Africa’s response to COVID-19

**DOI:** 10.1186/s12916-020-01622-w

**Published:** 2020-05-22

**Authors:** Chikwe Ihekweazu, Emmanuel Agogo

**Affiliations:** 1 Nigeria Centre for Disease Control, 801/4 Ebitu Ukiwe Street, Jabi, Abuja, Nigeria; 2 Resolve to Save Lives (Vital Strategies), Regus 4th Floor, Tower C, Churchgate Plaza, 473 Constitution Avenue, Central Business District, Abuja, Nigeria

**Keywords:** COVID-19, Africa, Response, Innovation, Public health

## Background

As the coronavirus disease (COVID-19) continues to spread, countries in sub-Saharan Africa are still experiencing outbreaks of other infectious diseases; the top causes of outbreaks from 2016 to 2018 were cholera, measles, and viral hemorrhagic diseases, such as Ebola virus disease, yellow fever, dengue fever, Lassa fever, and Rift Valley fever. These outbreaks have occurred alongside humanitarian crises and other public health emergencies in the region [1].

At the end of 2019, almost all African countries had undertaken a Joint External Evaluation (JEE) of the International Health Regulations (IHR). This process helped countries identify the gaps in their ability to prevent, detect, and respond to public health threats. Many countries had developed competencies in real-time surveillance and immunization, but overall, there was a pressing need for improving the resilience of the health sector in order to ensure effective outbreak response [2]. In response to recommendations from the JEEs, countries were supported to develop multisectoral National Action Plan for Health Security (NAPHS), addressing the gaps identified by the JEE and aligning with the various country sectoral plans.

This state of heightened alert across Africa has helped to prolong the containment phase of COVID-19 in many African countries. At the start of the pandemic in China, WHO’s African regional office and the Africa Centres for Disease Control provided guidance and technical and financial support to prepare countries. In our recent paper [3], we observed that Africa is better prepared than ever before because of our stronger national public health institutes, the rapid scale-up of testing capacity, better coordination at the continental level, and the capacity of built-in surveillance and contact tracing which has occurred since the 2013–2016 West African Ebola outbreak.

The first case of COVID-19 was reported in the African region on the 14th of February in Egypt, and in Sub-Saharan Africa on the 27th of February in Nigeria [3, 4]. This was over 1 month after the first case of the disease was reported in China, giving the region lead time to prepare for a large outbreak. As local transmission supersedes imported cases and the doubling time shortens to below 7 days in more than 95% of the affected countries [4], countries are now bracing for the impact of the pandemic.

Despite widespread misinformation about the immunity of Africans to COVID-19, overall poor health is driving mortality globally. Reports from other continents are showing higher morbidity and mortality in people of African heritage. While the observed disproportionality in adverse outcomes may be due to the impact of health disparities, poorer access to health care, and lower socioeconomic factors, the implication for African countries is clear.

At the end of April 2020, the continent has recorded a cumulative total of 20,652 cases and 861 associated deaths (case fatality ratio 4.2%) have been reported across 45 countries, the highest mortality have been recorded in Algeria 12.6% (425/3382), Liberia 9.7% (12/124), Democratic Republic of the Congo 6.1% (28/459), Mali 5.9% (23/389), Burkina Faso 6.6% (42/632), and Niger 4.2% (29/696) [3].

## Leveraging on Africa’s existing resources and innovation for COVID-19 response

African countries have focused on *intensive surveillance and case-finding*, leveraging the Integrated Disease Surveillance and Response framework (IDSR) [5], which all countries in the WHO AFRO region have adopted over the past 20 years. IDSR provides a framework for case-based and syndromic surveillance of forty conditions including influenza-like illness and severe acute respiratory illness. It provides an entry point for identifying, characterizing, and responding to community transmission of COVID-19.

The progressive *scaling up of molecular testing* across the continent has been impressive. Countries (such as South Africa and Nigeria) are leveraging and integrating molecular laboratory diagnostic capacity that exists for specific disease programs like drug-resistant tuberculosis, Lassa fever, and HIV, to scale-up testing for COVID-19. While access to diagnostic reagents becomes difficult, countries like Ghana are pioneering pooled testing of COVID-19 samples, therefore speeding up processing time and population coverage [6].

*Public health and social measures* have also been implemented across Africa. Countries have shut borders, introduced self-isolation for exposed persons, and established centers for quarantining of cases [3]. Countries and communities are on “lockdown.” As these public health measures flatten the rate of transmission, it is expected that the health system will have fewer cases of severely ill patients.

The gaps in service provision by the public health care system are being met by *private hospitals accredited by government authorities* to provide safe care. Access to advanced care in the public health system is limited; there are over 10 countries where there are few critical care beds, fewer experts with the training for managing critical patients with complex respiratory needs, and no ventilators [7]. Access to treatment, late presentation, and high levels of undiagnosed non-communicable diseases are likely to be the most important factors that will determine increased risk of mortality. Private health care providers are bridging the major gap that remains in the public health care sector to lead treatment facilities designated to provide care to the most critical patients.

In response to the lockdowns and the challenges of responding to the pandemic, *innovative use of technology and new ways of working* in health and other non-health sectors are being piloted. For instance, drones are being used to transport test kits and samples from hard-to-reach areas, thus reducing the sample transport time from many hours to minutes; there is a boom in locally manufactured face masks; an explosion of locally produced soap and hand sanitizers; and training, meetings, and workshops have moved online. Many governments have realized the need to improve hazard payments and provide insurance for staff on the frontline of the infection. African businesses have teamed up to donate in cash and kind to support country efforts, such as the $70 million donated by a coalition in Nigeria [8].

The *open, frequent, and transparent communication* of testing results within most African countries has kept the world abreast of the progress of the diseases despite the challenges of access to testing in many countries. The emergence of trusted voices and leadership by national public health institutes like the Nigeria Centre for Disease Control [9], the five regional Centres for Disease Control, and the apex Africa Centre for Disease Control after the West African Ebola outbreak [10] has added technical rigor and improved the quality of response by guiding political leaders to make better decisions on public health and social measures. Social influencers and celebrities have joined voices to public health experts urging people to practice social distancing.

## Conclusions

Despite the resilience of the people and some progress in public health systems, African countries will still be stretched as the pandemic spreads across the continent and as the containment measures succumb to the pressures of time, limited resources, and increased rate of infections. In the meantime, African countries are leveraging investments in syndromic surveillance and case-finding through IDSR; scaling molecular testing capacity developed for other diseases; deploying trainees in field epidemiology training programs to lead the field response; and using her most precious resource—her young people—to undertake contact tracing, staff isolation centers and provide the supportive care that is available. The management of these resources has shown to be the best fit in national public health institutes, which have been rapidly established and strengthened in the last decade in Africa.

So far, the response in Africa has been marked by *innovation* and *resilience* in the face of almost insurmountable odds and in the resounding collapse of multilateralism. However, as always, Africa will survive.

## Data Availability

Not Applicable.

## Abbreviations

COVID-19: Coronavirus disease
HIV: Human immunodeficiency virus
IDSR: Integrated Disease Surveillance and Response
IHR: International Health Regulations
JEE: Joint External Evaluation
NAPHS: National Action Plan for Health Security
